# A Safe Charging Algorithm Based on Multiple Mobile Chargers

**DOI:** 10.3390/s20102937

**Published:** 2020-05-22

**Authors:** Wei Wang, Haoran Jing, Junhua Liao, Feng Yin, Ping Yuan, Liangyin Chen

**Affiliations:** 1 School of Computer Science, Sichuan University, Chengdu 610065, China; wang.david.wei@stu.scu.edu.cn (W.W.); dt7133906@163.com (H.J.); jun_hua_liao@163.com (J.L.); 2 School of Computer Science and Technology, Southwest Minzu University, Chengdu 610041, China; yf_eagle@swun.edu.cn; 3 School of Mathematics and Information Engineering, Chongqing University of Education, Chongqing 400065, China; 13617659880@163.com; 4 Institude for Industrial Internet Research, Sichuan University, Chengdu 610065, China

**Keywords:** wireless sensor networks, safe charging, multiple mobile chargers

## Abstract

A safe charging algorithm in wireless rechargeable sensor network ensures the charging efficiency and the electromagnetic radiation below the threshold. Compared with the current charging algorithms, the safe charging algorithm is more complicated due to the radiation constraint and the mobility of the chargers. A safe charging algorithm based on multiple mobile chargers is proposed in this paper to charge the sensor nodes with mobile chargers, in order to ensure the premise of radiation safety, multiple mobile chargers can effectively complete the network charging task. Firstly, this algorithm narrows the possible location of the sensor nodes by utilizing the charging time and antenna waveform. Secondly, the performance of non-partition charging algorithm which algorithm allow chargers to charge different sensors sets in a different cycle is evaluated against the one of partition charging which does not allow for charging different ones. The moving distance of the charger node will be reduced by 18%. It not only improves the safety level which is inversely proportional to electromagnetic radiation but also expands the application scope of the wireless sensor nodes.

## 1. Introduction

Wireless sensor nodes widely exist in the Internet of things, these nodes are typically battery-powered and the amount of power they can use is relatively limited. Generally, most of the wireless sensor nodes are deployed outdoors, i.e., trees by the river, the roof of buildings and objects in the water. In these conditions, the battery cannot be replaced easily by us because of sensor nodes housed in a sealed waterproof case. As the development of the Internet of things, there are a trillion wireless sensor nodes all around the world while a million nodes need daily battery replacements. It is difficult and unrealistic to change batteries one by one in practical appliance. Therefore, how to prolong the network survival time has become an urgent problem and also a research hot area [1].

The current research mainly focuses on the following two aspects: (1) Energy saving. For example, nodes working in a low duty cycle mode maximize energy savings and prolongs the lifetime of the network by reducing the power consumption of sensor node [2]. In addition, some methods are scheduled according to the length of the data packet, so as to obtain a reasonable and effective energy management strategy [3]. However, the sensor data can not be uploaded to the sink node or the cloud server of the Internet of things in real-time. (2) Energy harvesting. Another way to solve the energy problem is powered from multiple low-level energy harvesting sources, including solar energy, wind energy, vibration energy, RF energy and more [4], especially in the usage of wireless power technology [5] in recent years. Currently, the emphasis has been placed on energy harvesting as the most important means of maintaining the wireless sensor network [1]. The Wireless Rechargeable Sensor Network (WRSN) has become a newly emerging research area of sensor network that utilizes RF charging as a convenient and efficient charging method [6] for sensor nodes or devices.

There is a great number of research works dedicated to wireless rechargeable sensor networks. Currently, the research directions of wireless rechargeable sensor networks are mainly divided into two aspects: on one hand, it is hardware which many researchers are trying to improve the efficiency of charging by the internal design of the sensor node and energy harvesting or wireless charging technology for improving the performance of the wireless rechargeable sensor networks; on the other hand, the researches focus on software improvement [7,8,9]. Some algorithms pay attention to optimizing the number of chargers, aiming to find the minimum number of charging nodes that satisfy the regional charging task, such as the algorithm in static scene [10] and the algorithm in dynamic scene [11], in order to improve the utilization of chargers. The other charging algorithms consider that the energy value of the sensor nodes needs to remain above a threshold to stabilize the operation. In order to reduce the charging delay, some algorithms for minimizing the charging delay are proposed, such as [12,13,14,15].

When most research resources were placed on the charging efficiency, there is a potential problem with the electromagnetic radiation exposure problem. In some literature, high electromagnetic radiation exposure has been recognized as a potential hazard to humans, including mental illness [16], tissue damage [17] and brain tumors [18]. There is also some tangible evidence that pregnant women and children are more vulnerable to high electromagnetic radiation [19]. For example, children’s heads absorb electromagnetic waves twice that of adults [20] under the same condition. In recent research, the radiation safety problem of wireless charging has begun to attract attention. Radiation safety charging algorithms in static scenes have been proposed, such as the Safe Charging for Wireless Power Transfer algorithm (SCWPT) [12], the Safe Charging with Adjustable PowEr algorithm (SCAPE) [21,22] and so on. The research scenario of these literature is that the chargers and sensor nodes are all stationary, and the radiation exceeding the standard value is avoided by controlling the switching sequence and time of each charger. The main difference is that SCWPT is based on the case where the charging voltage is variable, while SCAPE is based on the case where the voltage is not variable. Unfortunately, to the best of our knowledge, in the current algorithms, the safe charging algorithm is relatively few. The safe charging algorithm in the scene of static chargers is mainly studied. In this scenario, the charging node only responsible for charging some fixed number of sensor nodes has low usage efficiency and charging efficiency.

As the size of wireless sensor networks expands, multiple mobile chargers should be added and moved in the sensor networks [23,24]. However, safety problems have been taken into concern. In order to balance the relationship between charging efficiency and radiation safety in mobile conditions, this paper proposes a safe charging algorithm based on multiple mobile chargers.

The main contributions of this paper are as follows:

- The mobile chargers can find and charge the wireless sensor nodes quickly by using the charging time and antenna waveform.
- The non-partition charging algorithm is designed based on the solution of Multiple Traveling salesman Problem with a fixed depot, which is better than the performance of the partition one. In this algorithm, the performance of the chargers is fully utilized relative to partition charging one.
- This paper proposes a safe charging algorithm based on multiple mobile chargers, which can minimize total charge time and improve some performance, ensuring electromagnetic radiation below the safe threshold.

The remainder of the paper is organized as follows. In the Section 2, we express the basic model, node position correction method, and propose a safe charging algorithm based on multiple mobile chargers. Section 3 describes the details about the algorithm. Simulates and analyzes results are presented in the Section 4. Section 5 summarizes and discusses the future work.

## 2. Model and Algorithm

### 2.1. Basic Model

According to the Friis transmission equation [25], when electromagnetic wave propagates in space, the energy of wave attenuates with increased travel distance. The empirical model relationship of energy received and transmitted can be expressed as Equation (1):

(1) $$P_{r}\left(d\right)=\frac{\alpha }{{\left(d+\beta \right)}^{2}}$$

where, *d* is the actual distance between the sensor node and the charger, $\alpha =\frac{G_{s}G_{r}\eta }{L_{p}}{\left(\frac{\lambda }{4\pi }\right)}^{2}P_{t}$, β are the adjusted parameters in the short distance and $P_{t}$ is the transmitted energy of the chargers. If *D* is used as the farthest distance where the sensor nodes can effectively receive energy, the charging model can be expressed as the following Equation (2):

(2) $$P_{r}\left(d\right)=\left\{\begin{array}{cc} \frac{\alpha }{{\left(d+\beta \right)}^{2}}, & d\leq D\\ 0, & d>D \end{array}\right.$$

As the transmitted power increases, the intensity of the electromagnetic radiation also increases. The transmitted power and the intensity of the electromagnetic radiation have a positive correlation relationship. If *E* is used to indicate the intensity of electromagnetic radiation, *P* is the energy received by a sensor node, this model can be expressed as E(d)=k∗P(d), where *d* is the distance between the charging node and the sensor node, the constant *k* is the coefficient of the fitted line.

(3) $$E\left(d\right)=k*\sum_{i\in M}^{}P_{i}\left(d_{i}\right)$$

Assuming that the electromagnetic radiation density is also superimposed, the Equation (3) can be obtained, where *M* indicates that *M* chargers are working in the wireless rechargeable network, $d_{i}$ indicates the distance between the $i_{th}$ charger and the sensor nodes and E(d) indicates the electromagnetic radiation density generated by the final superposition of all chargers.

### 2.2. Node Position Correction

Since distance has a large influence on charging efficiency, the possible area of the sensor node should be further divided and narrowed. Therefore, the chargers can be closer to the sensor node to improve the charging efficiency. In this paper, the charging time and antenna waveform of sensor nodes are used for position correction.

#### 2.2.1. Localization Using Charging Time

**Figure 1:**
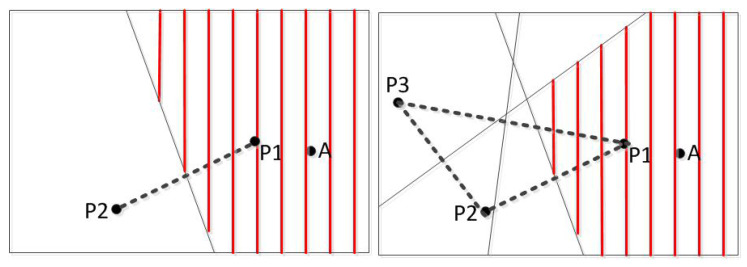
Schematic diagram of possible area segmentation.

As shown in the Figure 1, the location of a sensor node is *A*, and the charger charges the two positions $P_{1}$ and $P_{2}$ at the same time. With the same charge capacity, the charge time at $P_{1}$ is less than the one of $P_{2}$, so $P_{1}$ must be considered closer to *A* than $P_{2}$. Then it must be located on the near $P_{1}$ side of the vertical bisector of the $P_{1}$ and $P_{2}$ lines, which is the shaded area in the Figure 1. Assuming that another charge is made after these two charges and the charging position is $P_{3}$, there could be three wires between the three charging positions, and three vertical bisectors can also be generated, as shown in the Figure 1.

**Figure 2:**
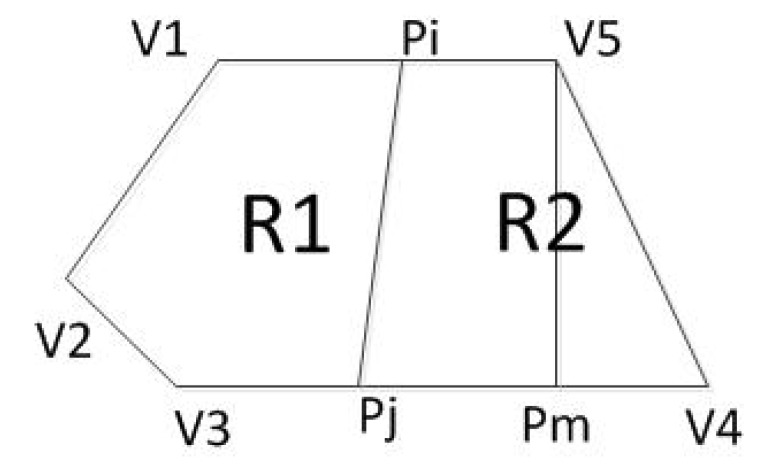
Schematic diagram of the possible area.

Based on the discussed above, assume that a possible region *R* of a sensor node calculated previously and its corner point set $v_{R}=\left\{v_{1},v_{2},v_{3},v_{4},v_{5}\right\}$, as in Figure 2, and an additional vertical line $l=(p_{i},p_{j})$ generated by a new charger stop position, where, $p_{i}$ and $p_{j}$ are the intersection point of *l* and the edges which belong to the possible location area *R*. In this type of positioning algorithms, the center of gravity of the possible location area is taken as the position of the node. The smaller the possible location area is, the more accurate node position will be. In the Figure 2, *R* is split into two sub-areas $R_{1}$ and $R_{2}$ after being split by the intersection line $l=(p_{i},p_{j})$. Since the diameter of the area is positively correlated with the size of the error, the optimal segmentation line is to minimize the diameter of the sub-area to obtain a more accurate node position. Firstly find the point set $p_{ij}$ and $p_{m}$, $p_{ij}$ satisfies $d\left(p_{ij},v_{i}\right)=d\left(p_{ij},v_{j}\right)$, $p_{m}$ satisfies $l\left(p_{m},v_{k}\right)⊥l\left(v_{i},v_{j}\right)$, where $d(p_{ij},v_{i})$ is the distance between line $p_{ij}$ and point $v_{i}$, and so to $d(p_{ij},v_{j})$. Calculate the distance between each point in the two point sets to all the corner points and find the two points with the smallest sum of the distances. The line connecting the two points is the optimal dividing line. The optimal segmentation line is the mid-perpendicular line of the current charging stop position and the optimal stop position. By this method, a better charging stop position [26] can be found.

#### 2.2.2. Localization Using Antenna Waveform

**Figure 3:**
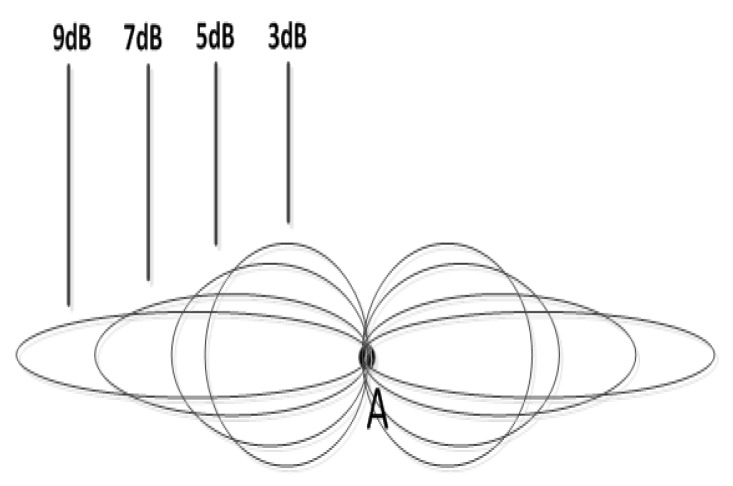
Schematic diagram of the possible area.

**Figure 4:**
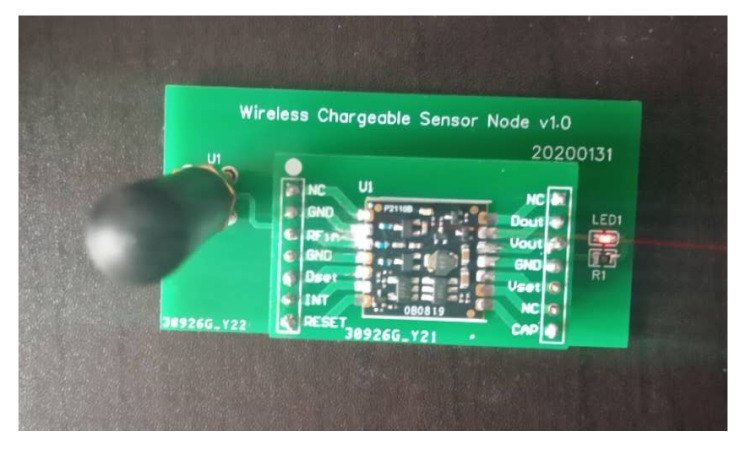
Pratical experimental sensor node.

**Figure 5:**
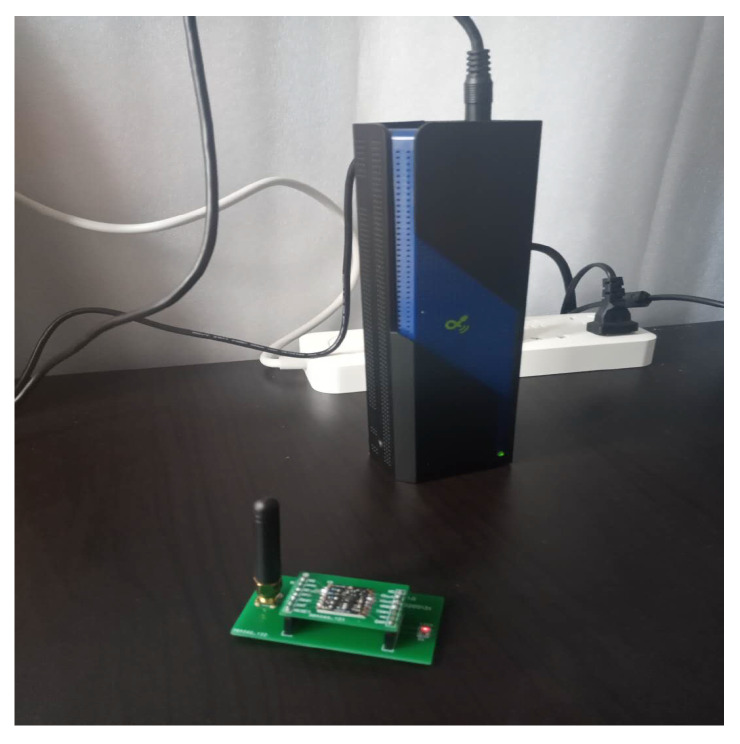
Experimental scene graph having the charger and wireless sensor node.

**Figure 6:**
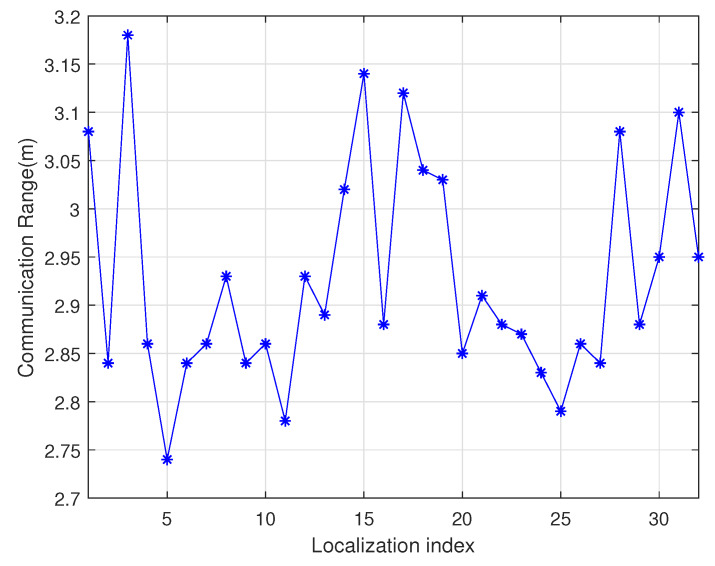
Sensor node communication range diagram.

**Figure 7:**
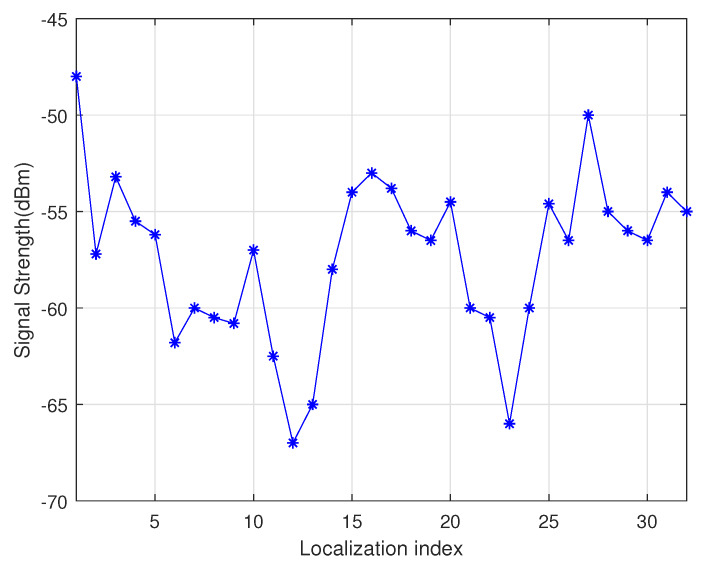
Sensor node charge signal strength.

As shown in Figure 3, the point *A* is the location of the charger, and the four adjacent rings are the communication coverage when the antenna gains are 3 dB, 5 dB, 7 dB and 9 dB, respectively. The size of the gain is inversely proportional to the wireless bandwidth. A typical sensor node uses an omnidirectional antenna below 9 dB, so the communication range is an approximate circle. To verify the above physical characteristics, the experiment uses the self-designed wireless rechargeable sensor node to divide the sensor node-centered direction into 32 parts and measure the communication range of the node in each direction as the Figure 4. The experimental scene is shown in the Figure 5, the wireless charger is the POWERCAST transmitter TX91503 and the sensor node is the self-designed PCB circuit board which has a led status for showing the received energy. The test results are shown in the Figure 6. It can be seen from the experimental results in Figure 6 that the communication range of the sensor node is not a complete circle, but an approximate circle. At the same time, a low antenna gain signal source is set outdoors, with the signal source as the center and the length of 3 m as the radius. The 32 points are measured at equal intervals on the circle, and the signal strengths of 32 positions are measured. The measurement results are shown in Figure 7. As shown in the figure, the signal strengths at different positions are different on the ring at the same distance from the signal source. The intensity of the signal has peaks and troughs, of which the trough is relatively obvious. Around the peak, there are more positions similar to the peak signal strength, but there are fewer similar signal strengths near the trough while the similar signal strengths are fewer near the trough. Since the signal source uses a low-gain antenna, its waveform is shown in Figure 7.

**Figure 8:**
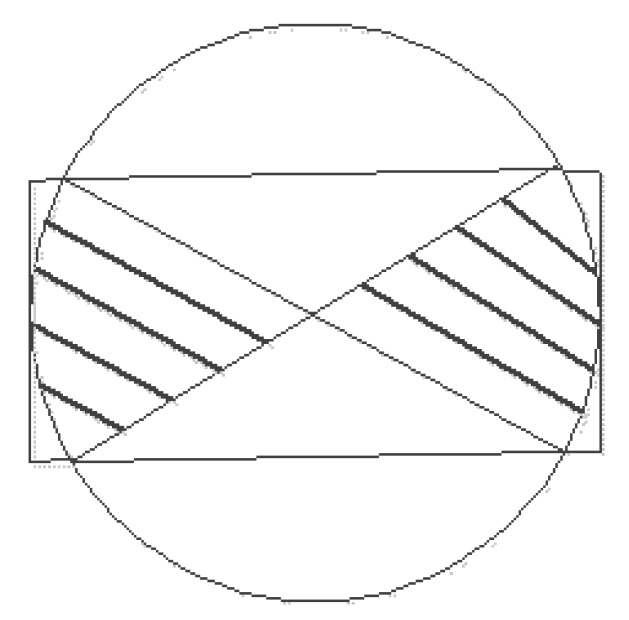
Charging coverage in rectangular area.

**Figure 9:**
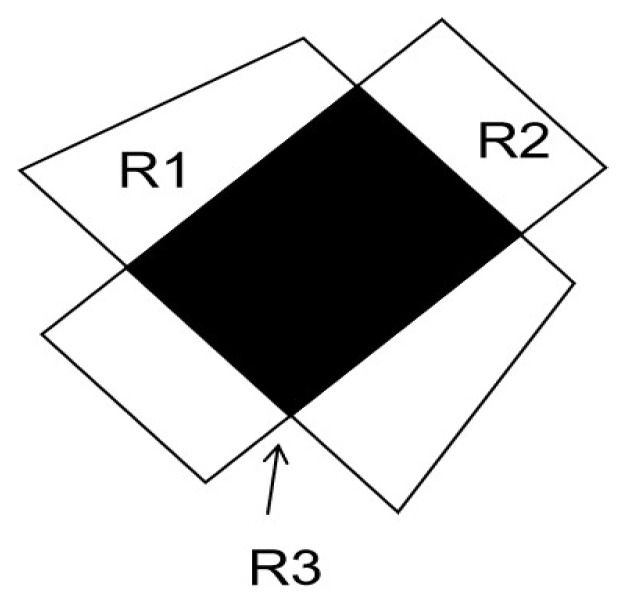
Charging coverage after waveform positioning.

The mobile chargers are carried by robots. When the chargers start charging at a certain position, it rotates at the center of its current position. Once a fixed angle is rotated, the sensor node records the signal strength correspondingly. The final sensor node will get signal strength data similar to the Figure 7. The sensor node notifies the charger of several stronger angles about the signal. When it turns these angles, the charger finds the direction in which the strongest position of the antenna signal is pointed. Then the sensor node is in the direction indicated by the charger. However it is difficult to determine the maximum value, so the largest part of the value is treated as the maximum value, thus a shadow area is formed as shown in the Figure 8. At the same time, in order to calculate easily, the rectangular area in the figure is used instead of the actual area for segmentation. This area is further superimposed with the existing possible areas to further narrow down the possible area. The positioning effect is shown in the Figure 9, where $R_{1}$ is the possible position area obtained by the previous positioning algorithm, and $R_{2}$ is the position after the waveform is used. The resulting area $R_{3}$ is the intersection of $R_{1}$ and $R_{2}$, which is the possible area of sensor node after waveform positioning.

### 2.3. Network Radiation Analysis

#### 2.3.1. Charging Area Discretization

**Figure 10:**
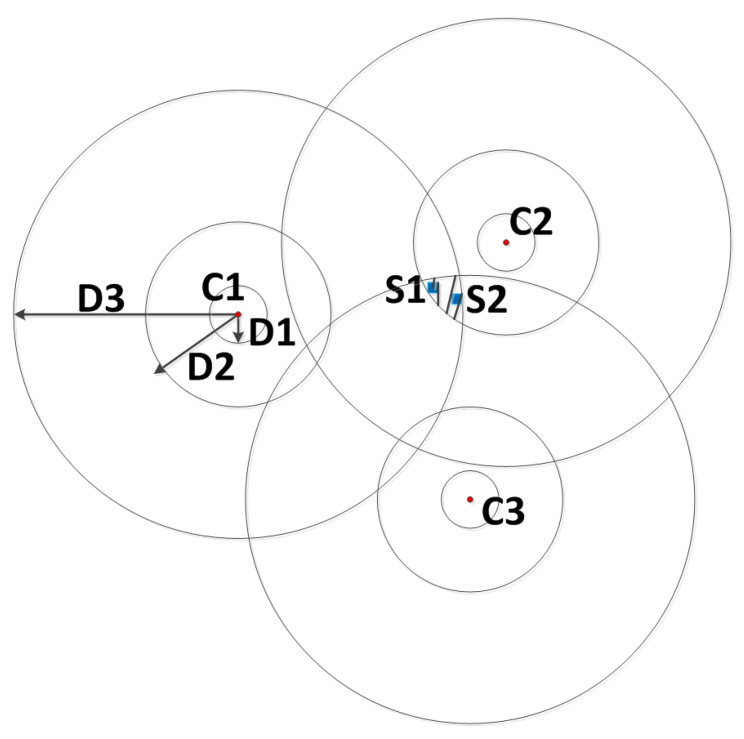
Charging area schematic.

For measuring the effect of a charger’s radiation on the environment, a large number of points should be measured. In order to solve or simplify this problem, when a robot with a charger rotates, the area to be charged by a charger can be divided by a discretization method. It converts the continuous values from 0 to *D* into a series of values. Suppose a charger covers an area in the range of 0 to *D*, centered on the location of the charger, using a set of concentric circles D[1], D[2], D[3] … D[G] divides the radiation area, and the change in energy value between concentric circles is fixed. *G* means that it is the number of the circles. Correspondingly, the energy of each ring after discretization is labeled as P[1], P[2], P[3] … P[G], and for each block is shown in Equation (4), where $c_{i}$ represents the $i_{th}$ charger, $k_{i}$ represents the *k* areas of the $i_{th}$ charger and $A_{c_{i}}\left(k_{i}\right)$ represents the energy value of the $k_{i}$ area of the $c_{i}th$ charger. For example, in Figure 10, G=3, at this time, the effective charging range of each charger is divided into three parts and the radiation intensity of the positions of the sensor nodes S1 and S2 can be considered to be the same. According to the step of discretization in the Figure 10, the shaded area can be expressed as $Ac_{1}\left(3\right)\cap Ac_{2}\left(2\right)\cap Ac_{3}\left(3\right)$. The higher the value of *G*, the more the number of concentric circles and the more accurate the calculation will be, with the higher computational complexity. Therefore, the selection of the *G* value needs to balance accuracy and time.

(4) $$P\left(k\right)=\cap_{c_{i}\in C}A_{c_{i}}\left(k_{i}\right),\;where,\;c_{i}\in \left\{c_{1},c_{2},\ldots ,c_{G}\right\}$$

#### 2.3.2. Radiation Analysis

When a sensor node receives energy from multiple chargers, it can be represented by the Equation (5). Correspondingly the radiation intensity can be expressed as the Equation (6). The distance from the charger to the sensor node is a constant between the charging power and the radiation intensity. Charging collection means that the charger in the collection is charging at the same time.

(5) $$P\left(s_{j}\right)=\sum_{j=1}^{M}P_{r}\left(d\left(s_{i},c_{j}\right)\right)$$

(6) $$E\left(s_{j}\right)=k\sum_{j=1}^{M}P_{r}\left(d\left(s_{i},c_{j}\right)\right)$$

(7) $$E\left(s_{j}\right)\leq R_{t}$$

All points in the coverage of all chargers need to be checked when we check radiation status. Discretize the area firstly and the radiation intensity values are calculated. By comparing the maximum radiance value with the threshold $R_{t}$, you can know if the radiation safety is met. If the radiation is safe, it can be charged directly.

### 2.4. Partition Charging Algorithm

Suppose the number of sensor nodes charged by a charger is $N_{x}$, and the number of nodes that need to be charged at the same time is $N_{s}$, where $N_{s}<N_{x}$. The partition charging algorithm is divided into two steps: (1) The charger in each partition plans its path according to request information and sorts ascending by path length. (2) Considering edge nodes in the algorithm and using the greedy algorithm to calculate the optimal global charging strategy.

#### 2.4.1. Edge Node Definition

**Figure 11:**
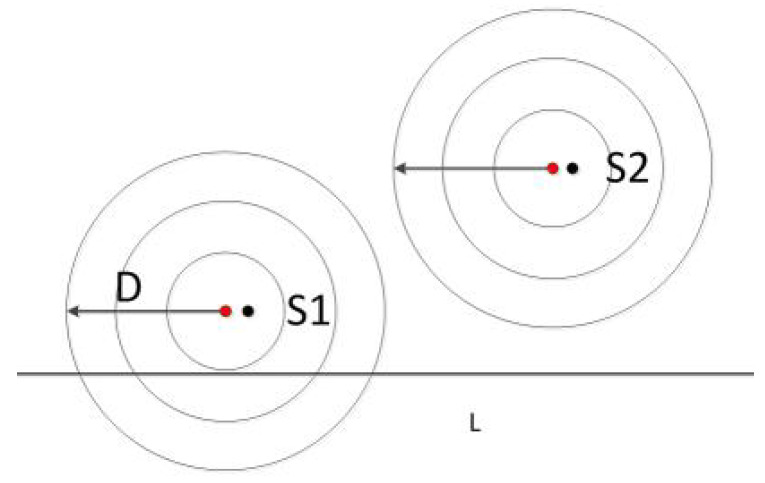
Edge node diagram.

When the charger is charging for a sensor node, the sensor node is defined as an edge node when the distance from the charger to the boundary of the partition is less than the charging effective distance *D*. As shown in the Figure 11, S1 is the edge node and the excessive radiation should be considered when charging. S2 is a non-edge node charged directly.

#### 2.4.2. Single Partition Path Plan

In the wireless rechargeable sensor network, there are *N* sensors to be charged, and other sensors are not considered and marked temporally. The charger calculates the deadline when each sensor starts charging and wait time for charging after arrival according to the request information. The charger should charge these sensors before the deadline of each one and the charger will plan a shortest path according to these limit values. Set deadline of the sensor $s_{i}$ to be $T_{i}$, the start charging time in the planning path to be $t_{i}$ and the distance for each node to be $d_{i}$, the path plan problem can be expressed as follows:

(8) $$\min\sum_{i=0}^{N_{x}}d_{i}\;s.t.\;T_{i}>t_{i},\;\left(i=1,2,3,\ldots ,N_{x}\right)$$

If there are $N_{x}$ nodes to be charged in the area, the charging path scheme has $A_{N_{x}}^{N_{x}}$. As a result of the deadline for every node, the options available will be greatly reduced. The charger lets only several possible schemes left and then sorts each scheme according to the length of the path from small to large. The reason why the optimal solution is not directly selected is that the optimal solution of a single region may not be the one entirely.

#### 2.4.3. Overall Area Path Plan

**Figure 12:**
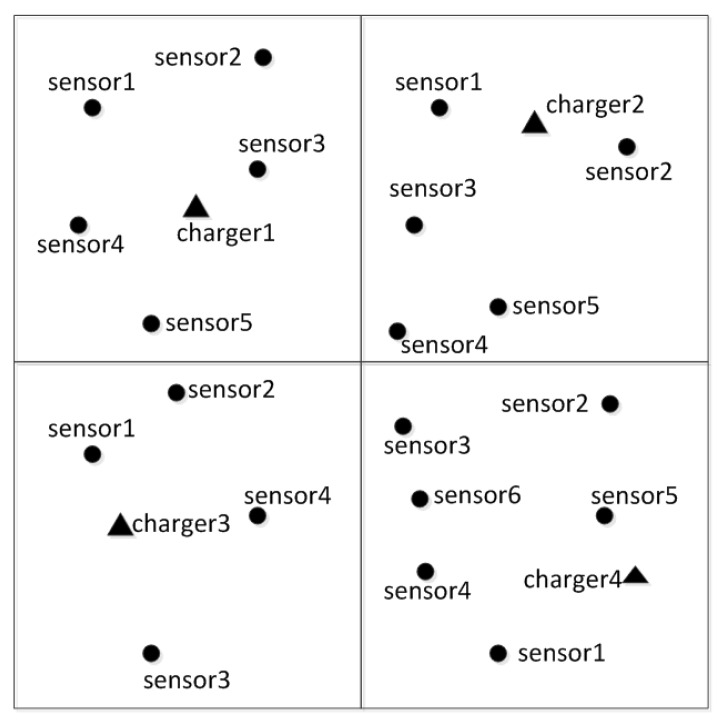
Overall area diagram.

The distribution of the overall area is shown in Figure 12. Each partition describes the charger and the sensors to be charged. Now we need to consider finding an optimal charging algorithm for the entire area comprehensively. In the algorithm for the whole region, obtain the optimal solution of each partition and calculate whether the edge node of the partition has a charging conflict. If the charging conflict occurs in some regions, the second optimal solution of each conflict region will be used instead of the optimal one. The solution is calculated to find the optimal solution that satisfies the overall safety conditions. If it is still impossible to avoid the charging conflict, the latter third optimal solution will be added for testing, until a solution that satisfies the safe condition has been selected.

### 2.5. Non-Partition Charging Algorithm

**Figure 13:**
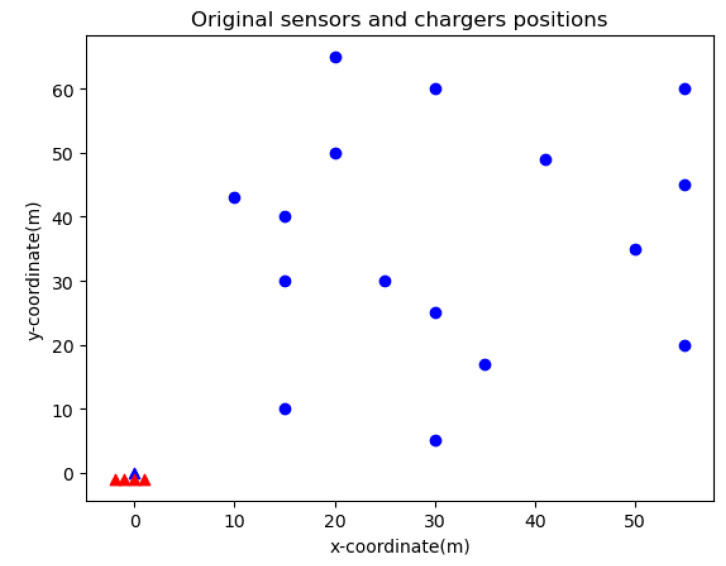
Schematic of non-partition.

In an overall area, there are *N* sensor nodes to be charged, *M* chargers. The nodes that do not need to be charged are independent of the algorithm, so they are not considered for the time being. As shown in the Figure 13, the dot in the figure is the node to be charged and the triangle is the charging node. Each charger is responsible for charging a certain number of sensor nodes and the sensor node should be safely charged before it expires.

#### 2.5.1. Charging Path Calculation

In order to find the optimal charge scheme, this problem can be converted into a multiple traveling salesman problem (MTSP) with a depot. In the literature [27], the variants of MTSP have been well defined and explained. The multiple traveling salesman problem consists of four subproblems and in this paper, one of them is solved, that is, finding the optimal charge scheme problem. Given a group of N wireless rechargeable sensors, the distance between any two sensors and M chargers starting from a fixed point, finding the nearly equal shortest tour for all chargers is our objective. The solving of MTSP is NP-hard, so the research of solvers is done by a few researchers all around the world which are used with some intelligent algorithms. In this paper, we solve MTSP-fixed with the heuristic algorithm by gurobi. According to the practical scene, the robots carried with the charger cost more energy and time in the moving action than that using for charging.

#### 2.5.2. Charging Path Allocation

**Figure 14:**
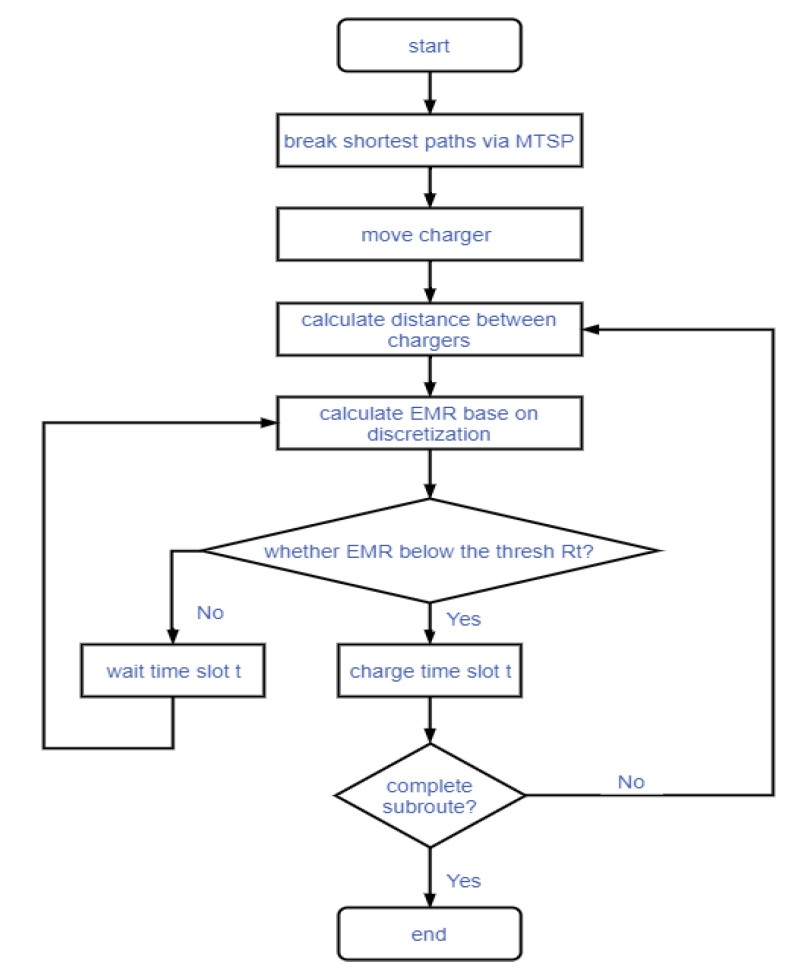
The flowchart of non-partition algorithm.

Among many results of SCBMC (Safe Charging Based on Multiple Mobile Chargers) algorithm based on MTSP-fixed, selecting the uniform distribution of subpath as the final path allocation. Considering the energy using the process of moving action is more than the energy using charging consumption for every node. In order to meet the challenge of nodes’ continual work, except for the timely charging work, the charging circuit or capacitor should keep the charging power above the threshold of $P_{max}$ in the duty cycle. Then when these chargers will charge for every node, firstly they judge the safety status in the neighbor area. After confirming the safety, the chargeable sensor nodes can request for charging. The entire safe charging process can follow the flow chart as shown in Figure 14.

## 3. Algorithm Descriptions

**Figure 15:**
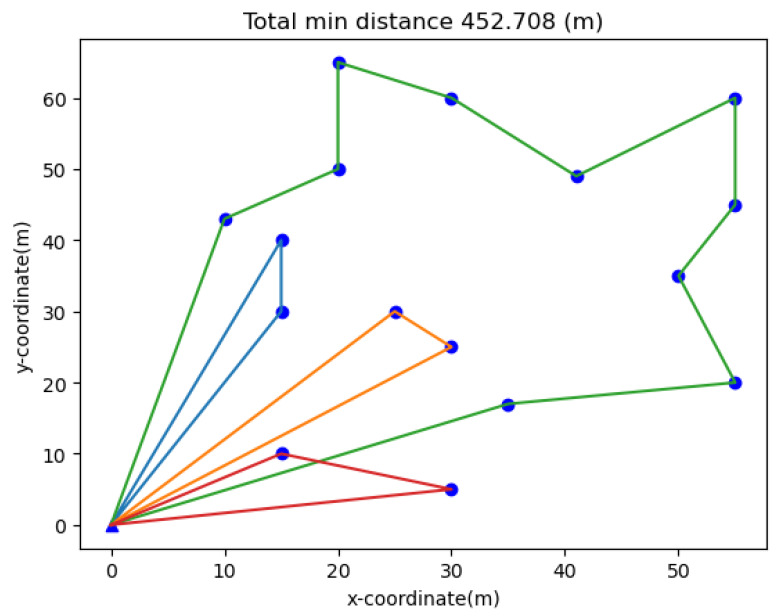
Schematic of sub route allocation.

The SCBMC algorithm proposed in this paper includes two steps, as the Algorithm 1. The first step is the MTSP-fixed algorithm for solving the optimal subroute allocation for the chargers. The second one is the charging decision and time consumption calculation step. In details, in the subroute allocation step, we can get the $i_{th}$ subroute, where i∈{1,2,3,4} in the Figure 15 for example. Therefore, the $i_{th}$ subroute belongs to the $i_{th}$ charger for moving later on. There are $N_{i}$ sensors marked as $s_{i,j}$, where $j\in \{1,2,\ldots ,N_{i}\}$. For simplification of computation, we assume that the waiting time slot and charging time slot is equivalent, both marked as *t*. The charging decision process should be acted according to the Equations (6) and (7). At last, the total delay time should be the last charger that has completed the subroute moving and charging tasks.
**Algorithm 1:** SCBMC algorithm based on MTSP-fixed.
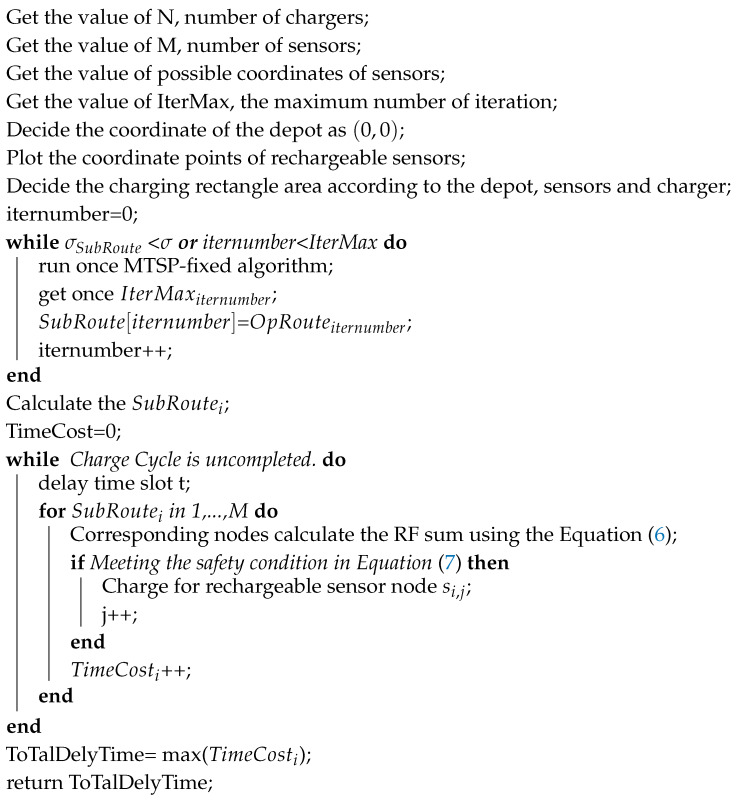


## 4. Simulation Test and Performance Analysis

In order to verify our algorithm, we test our framework by coding in python 3.7 importing the matplotlib, numpy and pandas packages. Gurobi [28] is used for solving the multiple travelling salesman problem. All tests are run on a desktop with a 1.8 GHz Intel i5-8250U processor and 8 GB of memory. Unless otherwise specified, the following default parameters setup is used in the simulation. It is supposed that there are 16 wireless rechargeable sensor nodes and 4 robots with chargers distributed in a 100 m × 100 m area. Regarding the charging model and EMR model, the EMR threshold is set to Rt = 125 μW/cm${}^{2}$. To simplify the experiment, the size of the waiting time slot and charging time slot are both set as 5 s.

### 4.1. Simulation Experiment Data Analysis

These main parameters are as follows:

Charging delay time: The time from charging request sending to being charged by the charger.

Charger moving distance: The distance traveled by the charger to charge sensor nodes during the movement in the network.

Total number of messages: The total number of information related to charging between the charger and the sensor node during the entire network operation.

#### 4.1.1. Performance Comparison with Sensor Node Number Change

**Figure 16:**
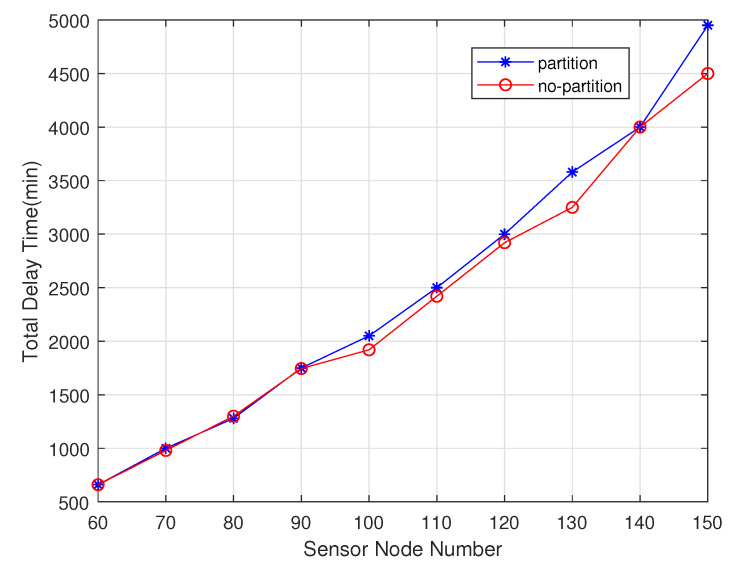
Total delay time varies with sensor nodes number.

**Figure 17:**
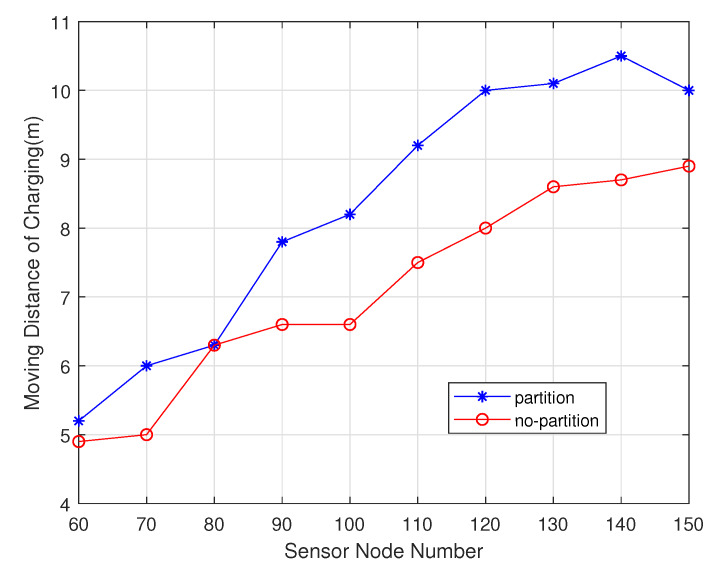
Moving distance of charger varies with sensor node number.

**Figure 18:**
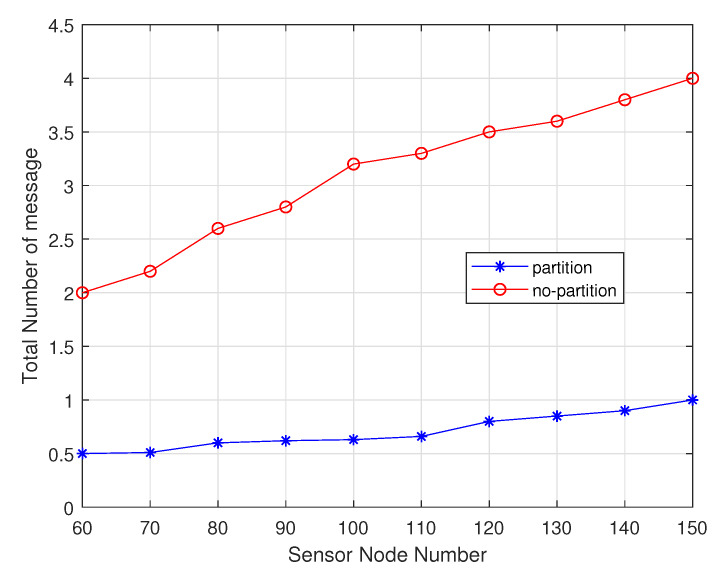
Total number of messages varies with sensor node number.

Among these experiments, the sensor nodes number gradually increases from 60 to 150. As shown in the Figure 16, non-partition algorithm can handle it due to the uneven energy consumption in different regions, so its charging delay is lower than the one of the partition algorithm. As shown in the Figure 17, the partition algorithm prefers the charger in this area to the non-partition algorithm, which tends to choose the nearest charging node, so The non-partitioned algorithm moves at a lower distance than the partition algorithm. As shown in the Figure 18, the node with non-partition algorithm needs to communicate with all chargers every charge, while the partition node only needs to communicate with the chargers it is responsible for in its area. The partition algorithm is better than the non-partition algorithm.

#### 4.1.2. Performance Comparison with Chargers Number Change

**Figure 19:**
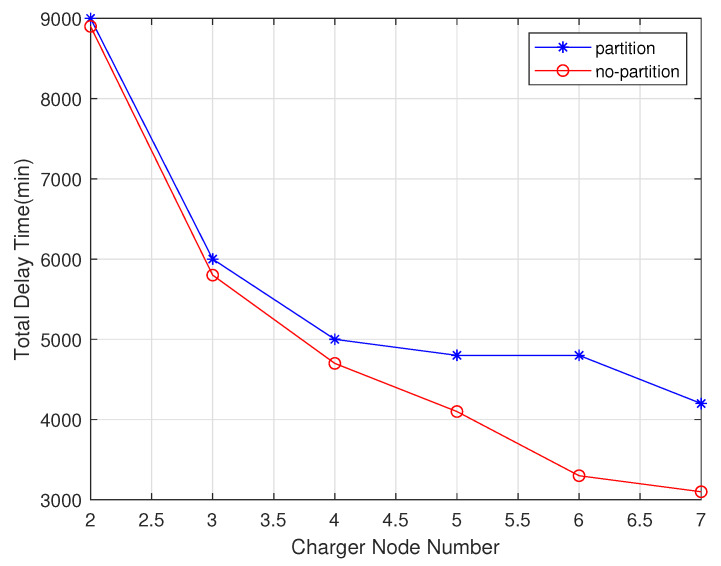
Total delay time varies with charger number.

**Figure 20:**
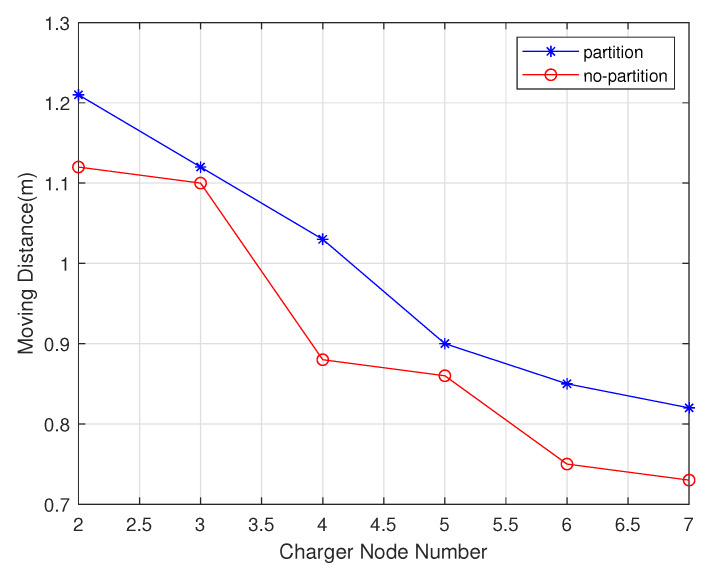
Charger node distance varies with charger number.

**Figure 21:**
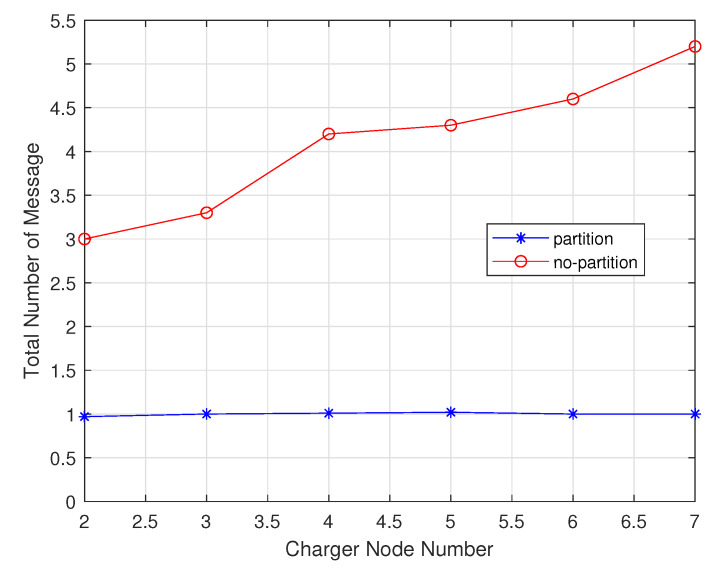
Total message number varies with charger number.

In these experiments, the chargers number increased from 2 to 7. As shown in the Figure 19, the number of chargers is negatively correlated with total delay time. The charging delay of the non-partition algorithm is lower than that of the partition algorithm. As shown in the Figure 20, since the partition algorithm will select the charger as much as possible rather than the non-partition algorithm, it will tend to choose the nearest charger, so the moving distance of non-partition algorithm is lower than the partition algorithm. As shown in the Figure 21, since the non-partition algorithm needs to communicate with all chargers for each charging, the partition algorithm is superior to the non-partition algorithm.

### 4.2. Comparison between SCBMC, SCWPT and SCAPE

**Figure 22:**
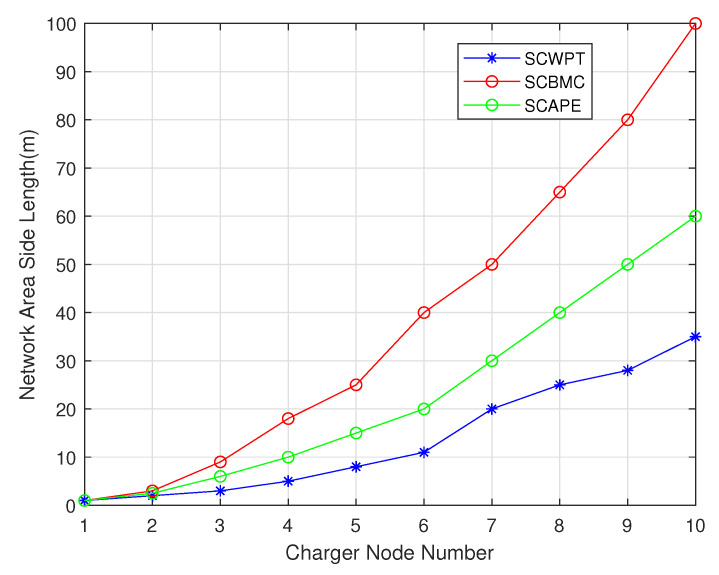
Comparison between SCBMC, SCWPT and SCAPE about network area expansion.

**Figure 23:**
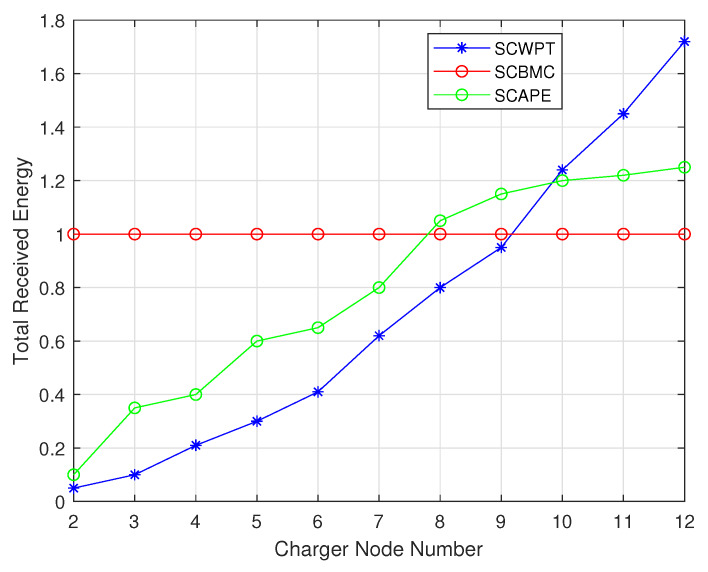
Comparison between SCBMC, SCWPT and SCAPE about total received energy.

The main difference between SCBMC, SCWPT and SCAPE algorithm in the scene include charging methods, mobility and distribution density. The different charging methods make the energy utilization different, calculated according to the Equation (2). Since the SCBMC algorithm selects the sensor node near the charger for charging, charging efficiency is relatively stable. While the SCWPT algorithm has lower charging efficiency when there are fewer nodes in the area. As node number increases, the charging efficiency increases gradually. When there are 10 sensor nodes, the charging efficiency is higher than the SCBMC algorithm. It can be seen that SCWPT is suitable in high density sensor networks. When the density is low, the SCBMC algorithm is more efficient. With the decreasing of the density of sensor nodes, the SCWPT algorithm will face unusable conditions, while the SCBMC algorithm can be used at any node density. From the Figure 22 and Figure 23, as the sensor network area expands, chargers number increases in these algorithms. The growth rate of the SCWPT and SCAPE algorithm is faster than that of the SCBMC algorithm and the number of chargers required in a large-scale network is more. The SCBMC algorithm can cover a larger area because the charger can move, so when the network area increases, the number of required chargers grows slowly.

**Figure 24:**
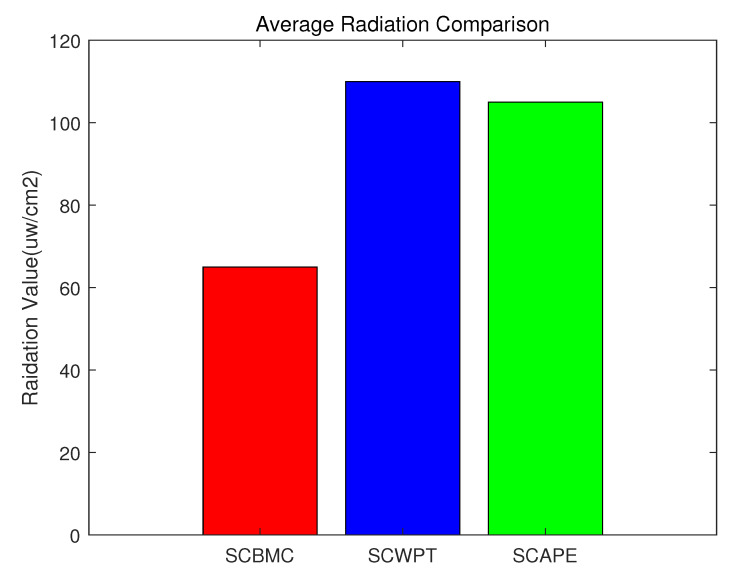
Comparison between Safe Charging Based on Multiple Mobile Chargers algorithm (SCBMC), Safe Charging for Wireless Power Transfer algorithm (SCWPT) and Safe Charging with Adjustable PowEr algorithm (SCAPE) about average radiation.

Figure 24 depicts the radiation metric for each algorithm. As we see, the radiation safety level of the SCBMC algorithm outperforms that of the SCWPT algorithm and the SCAPE algorithm. The waiting time slot and charging time are both the same, therefore the average radiation value of the SCBMC algorithm usually equal to half of the EMR threshold. According to the literature of SCWPT and SCAPE, the radiation values of them are just a little bit below the threshold. In details, the radiation value of the SCBMC is 65 μW/cm${}^{2}$, however, the radiation value of the SCWPT is 110 μW/cm${}^{2}$ and the radiation value of the SCAPE is 105 μW/cm${}^{2}$. At the same time, ensuring the safety threshold value, the total moving distance of chargers can be kept to a minimum.

In this subsection, the robustness against the different number of the chargers and sensors about the proposed methods is tested on simulation programming implemented in python. The gurobi solver can solve the unique solution in a relatively short period of time. At the same time, for simplification in computation, the waiting time and charging time have been set to slot *t*. Therefore, the unique solution for the minimum total distance also ensures the robustness of the algorithm.

## 5. Discussion

The background of wireless charging technologies has been reviewed extensively first, and then with the boom of the wireless network, the safety problem about charging has been taken into consideration. The classical models of energy transmission have been modeled by us. Use the charging time and the antenna waveform to further segment and narrow the possible location of the node to improve the charging effectiveness. This paper proposes a safe charging algorithm for wireless rechargeable sensor networks under multiple mobile chargers. This type of charging problem is converted into a MTSP-fixed problem and the subroute of every charger can be solved robustly. After the confirmation of the subroute, every charger move, wait and charge the rechargeable sensors along the way of subroutes which ensure that the EMR value is not greater than the healthy level. The simulation results show that the SCBMC algorithm has better charging effectiveness than others below the safe threshold.

However, there are some limitations to our proposed SCBMC algorithm. The first limitation of SCBMC is that most of it is performed in only simulations. In the future, we will establish a test-bed to verify all the simulations. The second main limitation of SCBMC is that we suppose the charging area discretization. In the future, we will explore a more fine-grained discretization method for EMR. The third limitation of SCBMC is that we do not consider a longest delay minimization problem for sensor charging under multiple chargers because this is another NP-hard problem. In the future, we will investigate a novel safety algorithm that meets this condition.
